# Impact of immunopathology on the antituberculous activity of pyrazinamide

**DOI:** 10.1084/jem.20180518

**Published:** 2018-08-06

**Authors:** Landry Blanc, Jansy Passiflora Sarathy, Nadine Alvarez Cabrera, Paul O’Brien, Isabela Dias-Freedman, Marizel Mina, James Sacchettini, Radojka M. Savic, Martin Gengenbacher, Brendan K. Podell, Brendan Prideaux, Thomas Ioerger, Thomas Dick, Véronique Dartois

**Affiliations:** 1 Public Health Research Institute, New Jersey Medical School, Rutgers, The State University of New Jersey, Newark, NJ; 2 Department of Biochemistry and Biophysics, Texas A&M University, College Station, TX; 3 Department of Bioengineering and Therapeutic Sciences, Schools of Pharmacy and Medicine, University of California, San Francisco, San Francisco, CA; 4 Department of Microbiology, Immunology and Pathology, Colorado State University, Fort Collins, CO; 5 Department of Computer Science, Texas A&M University, College Station, TX; 6 Department of Medicine, New Jersey Medical School, Rutgers, The State University of New Jersey, Newark, NJ

## Abstract

In the 1970s, inclusion of pyrazinamide (PZA) in the drug regimen of tuberculosis (TB) patients for the first 2 mo achieved a drastic reduction of therapy duration. Until now, however, the mechanisms underlying PZA’s unique contribution to efficacy have remained controversial, and animal efficacy data vary across species. To understand how PZA kills bacterial populations present in critical lung lesion compartments, we first characterized a rabbit model of active TB, showing striking similarities in lesion types and fates to nonhuman primate models deemed the most appropriate surrogates of human TB. We next employed this model with lesion-centric molecular and bacteriology readouts to demonstrate that PZA exhibits potent activity against *Mycobacterium tuberculosis* residing in difficult-to-sterilize necrotic lesions. Our data also indicate that PZA is slow acting, suggesting that PZA administration beyond the first 2 mo may accelerate the cure. In conclusion, we provide a pharmacodynamic explanation for PZA’s treatment-shortening effect and deliver new tools to dissect the contribution of immune response versus drug at the lesion level.

## Abstract (precis)

This study presents a comprehensive lesion-centric analysis of disease progression in the rabbit model of tuberculosis, showing immunopathology and lesion heterogeneity similar to human and nonhuman primates. In this model, pyrazinamide sterilizes necrotic lesions, where persistent bacterial populations reside.

## Introduction

With the advent of positron emission tomography/computed tomography imaging to monitor disease progression and therapeutic response in tuberculosis (TB) patients and nonhuman primates as well as rabbits, it has become apparent that within the same host different lesions can follow diverging trajectories in response to drug treatment (Via et al., 2012; Coleman et al., 2014; Malherbe et al., 2016). Clinical data accumulated over several decades indicate that the presence and extent of cavitary disease is associated with treatment failure and relapse (Aber and Nunn, 1978; Chang et al., 2004, 2006) or inferior outcome (Savic et al., 2017), pointing to necrotic cavities as a key lesion type driving persistence of TB disease. Likewise, in preclinical efficacy studies that assess bacterial populations surviving drug treatment, the lesion compartments that fail to be sterilized at the end of therapy are mostly the caseous foci of closed nodules and cavities (Lenaerts et al., 2007; Lin et al., 2009; Hoff et al., 2011; Via et al., 2015a). Thus, targeting lesions with a necrotic component appears critical to achieve treatment shortening and durable cure.

Pyrazinamide (PZA) was discovered in the 1950s and rapidly demonstrated clinical utility when added to TB drugs available at the time (Fox et al., 1999). Together with rifampicin, PZA contributed to dramatic therapy shortening from 18 to 6 mo while maintaining low relapse rates (Hong Kong Chest Service/British Medical Research Council, 1982; Fox et al., 1999). As a result, PZA has been part of today’s first-line TB drug regimen since the 1980s and is also used to treat most multidrug-resistant cases. Based on numerous experiments in mice, this treatment-shortening property is attributed to PZA’s ability to eliminate persisting bacterial populations (McCune et al., 1956; McCune and Tompsett, 1956) and achieve apparent sterilization in different mouse models (Lanoix et al., 2016 McCune et al., 1966a,b; Williams et al., 2012). Although early clinical trials failed to detect a benefit of prolonging PZA treatment beyond the first 2 mo, mouse efficacy data suggest otherwise in the background of both first- and second-line regimens (McCune et al., 1966b; Ahmad et al., 2013; Lanoix et al., 2016).

Because PZA is largely recognized as a “sterilizing” drug (Mitchison, 1985, 2004b; Chigutsa et al., 2015) and necrotic lesions are most recalcitrant to therapy, it follows that PZA should be efficacious against bacterial populations that inhabit necrotic niches. However, direct demonstration of PZA’s activity against these populations is lacking, and several recent findings indirectly support or contradict this hypothesis (Ahmad et al., 2011; Irwin et al., 2015; Lanoix et al., 2015a; Prideaux et al., 2015; Kempker et al., 2017; Sarathy et al., 2018). Interestingly, we previously observed that PZA contributes very little if anything in reducing bacterial burden in C3HeB/FeJ mouse necrotic lesions (Lanoix et al., 2015a; Irwin et al., 2016). To test the killing potential and sterilizing activity of PZA in specific lesion types, we resorted to the rabbit model of active TB, known to exhibit a spectrum of human-like lesions (Manabe et al., 2003; Subbian et al., 2011; Via et al., 2012). First, the model was extensively characterized to assess the heterogeneity of individual lesion trajectory with regard to bacterial burden and immune-mediated killing by using lesion-centric bacteriologic and molecular readouts that quantify both bacterial burden (measured as CFUs) and cumulative burden of viable and dead bacteria (measured as chromosome equivalents [CEQs]; Muñoz-Elías and McKinney, 2005; Lin et al., 2014). In TB patients and nonhuman primates, the mechanisms that establish diverging granuloma fates involve complex interactions among host immune responses, inflammation, and bacterial processes (Sukumar et al., 2014; Marakalala et al., 2016; Cadena et al., 2017). Indeed, lesion-centric analyses in rabbits with active TB revealed that, although some lesions seem to restrict bacterial growth and achieve local sterilization, others display increasing bacterial burden as disease progresses. These observations indicated that immunopathology and lesion heterogeneity compare favorably between rabbits and nonhuman primates or TB patients (Coleman et al., 2014; Lenaerts et al., 2015; Martin et al., 2017). We next built on these findings to measure PZA-mediated killing at the lesion level. Our results validate the rabbit model as a tool to evaluate TB drug efficacy. They also provide an incentive to revisit the use of PZA beyond the 2-mo intensive phase of therapy and to discover more-potent PZA analogues to accelerate the cure and reduce relapses of a disease that recently became the single leading infectious cause of death in adults, surpassing HIV (World Health Organization, 2017).

## Results and discussion

### Heterogeneity of lesion trajectory in the rabbit model

In TB patients and nonhuman primates, pulmonary lesions are characterized by diverging trajectories both in terms of bacterial load and immunopathology (Coleman et al., 2014; Martin et al., 2017). To compare the rabbit model of active TB to human disease and nonhuman primate models and assess its utility as a tool to measure drug efficacy in different lesion types, we performed lesion-centric analyses over the course of 20 wk of chronic disease. This initial analysis included 40 rabbits and 1,063 lesions. Individual lesions were subjected to bacterial burden quantification (CFU) and histopathology profiling at various time points from 4 to 20 wk postinfection. In addition, cumulative burden was measured by real-time quantitative PCR (qRT-PCR) to capture total viable and dead bacilli as CEQs and infer the efficiency of killing by the immune response in each lesion.

**Figure 1.**
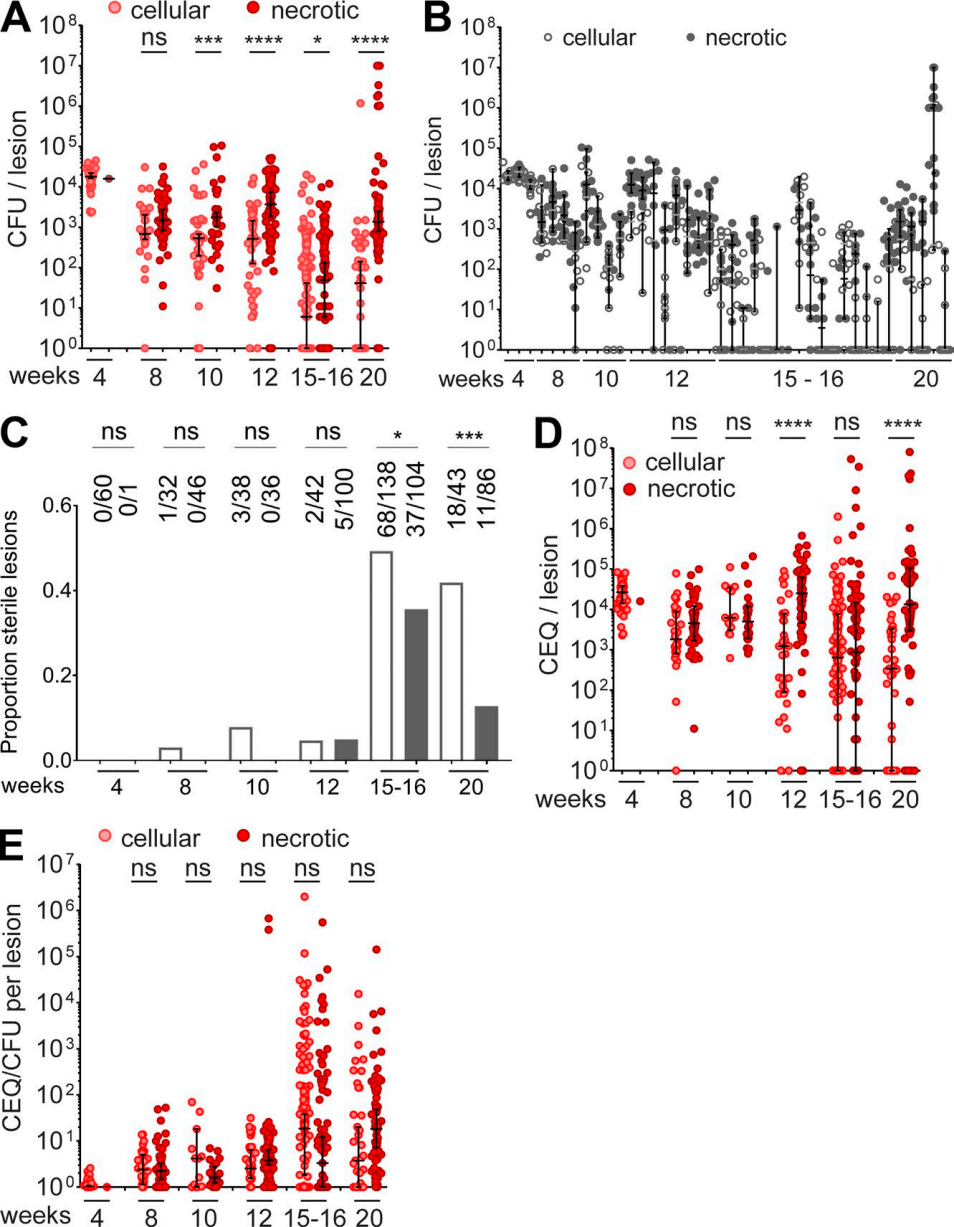
**Lesion-centric bacterial burden and cumulative burden in drug-naive rabbits with active TB. (A)** Evolution of bacterial load per lesion from 4 to 20 wk postinfection. **(B)** Range of CFU/lesion within individual rabbits from 4 to 20 wk postinfection. **(C)** Proportion of cellular (empty bars) and necrotic (gray bars) lesions as disease progresses from 4 to 20 wk postinfection; actual numbers of sterile versus total lesions are indicated on top of each bar. **(D)** Evolution of the cumulative bacterial burden per lesion from 4 to 20 wk postinfection. **(E)** CEQ/CFU ratios within lesions, an indirect measure of the extent of immune-mediated killing, over the 20-wk disease course. Data from three independent experiments were combined to generate the plots. Asterisks indicate statistical significance by using the Mann-Whitney *U* test in A, B, D, and E and Fisher’s exact test in C. *, P < 0.05; **, P < 0.01; ***, P < 0.001; ****, P < 0.0001.

To track lesion burden over time in drug-naive animals, we plotted the CFU in cellular and necrotic lesions (where “necrotic lesions” encompass caseous granulomas and cavities) over the entire 20-wk course of infection (Fig. 1 A). At 4 wk postinfection, all but one of the granulomas were cellular with a relatively uniform bacterial load, slightly >10^4^ CFU per lesion. From then on, lesion burden started to diverge and reached the widest range at 20 wk postinfection, from fully sterile (no recoverable CFU on agar with a limit of detection [LOD] of 5 CFU per lesion) to 10^7^ bacilli per lesion. In rabbits with established TB disease (12­–20 wk postinfection), we found that the bacillary burden per lesion was highly variable within individual animals (Fig. 1 B). Across time points, necrotic granulomas and cavities harbored higher bacterial burden than cellular lesions. We did not observe any lung lobe bias in the distribution of lesions and bacterial burden (not shown). Sterile lesions started to appear around 8–10 wk, and the bulk of lesion sterilization was achieved between 12 and 15 wk postinfection (Fig. 1 C). At 15 and 20 wk postinfection, the proportion of sterile lesions was significantly higher in the cellular granuloma category (Fig. 1 C), with 49 and 35% in the cellular category and 42 and 13% in the necrotic category, respectively. Similar proportions have been observed in drug-naive nonhuman primates (Lin et al., 2014). As observed for the range of CFUs per lesion, the range of lesion mass within individual rabbits was tight early on and increased as disease progressed (Fig. S1 A). A correlation trend was observed between bacterial load and lesion weight (r^2^ = 0.49 and 0.38 for cellular and necrotic lesions, respectively; Fig. S1 B). We also quantified the burden of seemingly uninvolved lung (Fig. S1 C, lung parenchyma with various degrees of cellular infiltration by lymphocytes, histiocytes, and polymorphonuclear cells, not organized as circumscribed lesions). We found a substantial bacterial burden in uninvolved lung, the median of which was approximately two orders of magnitude lower than in lesions up to 12 wk postinfection (Fig. S1 D). This low burden could originate from microlesions not detected upon macroscopic examination and dissection or from immune-privileged sites where small numbers of *Mycobacterium tuberculosis* (Mtb) bacilli are “tolerated” by the immune system. At 4 wk, all uninvolved lung samples were CFU-positive (median 2.2 log CFU per lesion), similar to cellular lesions (median 4.2 log CFU per lesion). As disease progressed, uninvolved lung followed the same sterilization pattern as cellular lesions (Fig. S1 D), compatible with the notion that the burden present in uninvolved lung could originate from small cellular lesions, which the immune system either eradicates or allows to develop as mature lesions. This doesn’t exclude the possibility that uninvolved lung also contains immune-privileged sites that harbor pathogen reservoirs. One could speculate that “dominant” lesions attract the bulk of immune cells, causing spatial and temporal relief of immune pressure in neighboring areas.

To assess the degree of bacterial killing in individual lesions, we took advantage of the finding that genomes (CEQs) from nonviable Mtb largely persist in tissues (Muñoz-Elías et al., 2005; Lin et al., 2014). CEQs reflect the cumulative bacterial burden, and the CEQ/CFU ratio is an indirect measure of bacterial killing over time. Similar to CFU, the cumulative burden per lesion fell within a tight range at 4 wk, after which the range widened dramatically to peak between 15 and 20 wk postinfection (Fig. 1 D), with a high within-animal variability in CEQ/lesion after 8–10 wk (Fig. S1 E).

Compiling the CEQ/CFU ratios per lesion over time revealed virtually no kill (median CEQ/CFU = 1) at 4 wk postinfection. From then on, lesion trajectories started to diverge both in cellular and necrotic lesion categories (Fig. 1 E). The range of CEQ/CFU ratios markedly increased between 12 and 15 wk, consistent with the concomitant burst in sterile lesions (Fig. 1 C). As CFUs increased, the spread of CEQs decreased, suggesting failed immunity in lesions with high bacterial burden (Fig. S1 F). Accordingly, we found an inverse correlation between the CEQ/CFU ratio and the CFU content of individual lesions, from completely sterile with high CEQ/CFU to very high bacterial burden with poor killing (Fig. S1 G). Separate plots of CEQ versus CFU at each time point showed that departure from the CFU-CEQ line of equality increases over time, until week 16 when immune mediated killing appears to peak, and that failure of the immune response in a subset of the lesions was best revealed from week 16 to week 20, where high-CFU lesions (>10^6^ CFU per lesion) appeared with little to no killing (Fig. S1 H). Altogether, these observations demonstrated diverging lesion trajectories reminiscent of the nonhuman primate model of active TB (Lin et al., 2014; Via et al., 2015a), thus confirming the utility of the rabbit model to study the lesion-centric effect of TB drugs.

**Figure 2.**
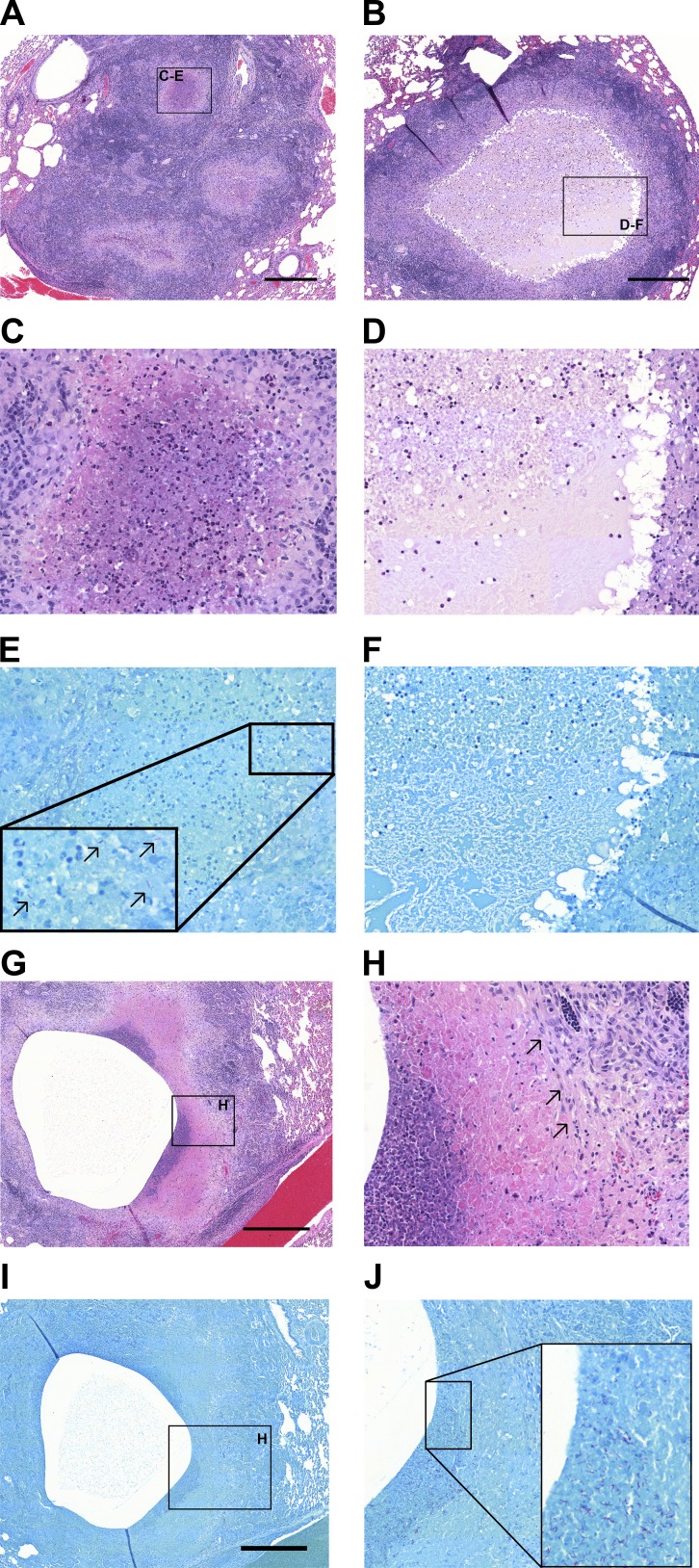
**Histopathological heterogeneity in rabbits with active TB and association of Mtb bacilli with neutrophils. (A)** H&E staining of a cluster of small necrotizing granulomas with extensive infiltration of polymorphonuclear neutrophils in the nascent necrotic core, surrounded by a rim of epithelioid macrophages and lymphocyte-dominated inflammation at the periphery (16 wk postinfection). **(B)** Large necrotic granuloma with classical eosinophilic caseum in the necrotic core and low cellularity consisting of few degenerate neutrophils (16 wk postinfection). Foamy macrophages are located adjacent to the caseous necrosis. Panels C and E and panels D and F show a magnification of the regions highlighted in A and B, respectively. **(C)** Active neutrophil influx in the early necrotic core. **(D)** Caseum with limited cellular infiltrate. E and F are Ziehl-Neelsen stains of the regions shown in C and D. Black arrows in E point to Mtb bacilli found in high numbers in areas with high neutrophilic infiltration. **(F)** Lack of acid-fast bacilli within the paucicellular caseum. **(G)** H&E staining of a large cavity at 8 wk postinfection shows prior expulsion of central necrotic material and inflammatory destruction and erosion into neighboring pulmonary vein. **(H)** Magnification of the neutrophil-rich region highlighted in G showing the interface between low- and high-cellularity areas with high density of neutrophils. Black arrows point to an area of fibrosis at the cavity margin. **(I and J)** Ziehl-Neelsen stains of the regions shown in G and H, with high numbers of bacilli found in the vicinity of neutrophils. Scale bars: 500 µm.

Histopathologic analyses revealed mostly cellular granulomas up to 4 wk (1–4 mm in diameter), at which point the pathology diverged, leading to the presence of cellular, early-necrotizing and fully necrotic granulomas of various sizes (ranging from 1 to 10 mm) from 10 wk onward, as reflected by the increase in the range of lesion mass within individual rabbits (Fig. S1 C). Clusters of coalescing granulomas and occasional cavities were observed after 8 wk, and lesion size exceeded 10 mm in rare occasions (up to 25 mm). A minority of rabbits presented with mild pathology and very few organized lesions. The cellularity of necrotic foci in granulomas and cavities was highly variable and mostly composed of neutrophils (Figs. 2 and S2). Acid-fast staining revealed a higher abundance of bacteria in caseous foci with higher neutrophilic infiltration, aligned with previous observations that neutrophils appear to create a nutrient-rich environment and that Mtb exploits neutrophilic inflammation to preferentially replicate at sites of tissue damage that promote transmission (Mishra et al., 2017; Philips, 2017; Figs. 2 and S2). We measured the pH of the caseous foci of ∼20 lesions and found a range of values from 6.1 to 8.0 (median, 6.7), reflecting an additional dimension of rabbit lesion heterogeneity (Table S1).

To assess genetic heterogeneity of Mtb at the lesion level, we subjected 23 strains isolated from 23 lesions (cellular and necrotic from four rabbits infected for 20 wk) to whole-genome sequencing. Five strains (22%) displayed one to three single nucleotide polymorphisms (SNPs), and different polymorphisms were found in different lesions within the same animal (Table S2), indicating localized genetic variation. In total, we detected nine SNPs in 3,220 lesion days (23 lesions from rabbits infected for 140 d) or one SNP per 358 lesion days. In comparison, Ford et al. (2011) detected four SNPs in 1,541 nonhuman primate lesion days, or one SNP in 385 lesion days. Thus, bacterial variability is similar in both models and supports the concept of lesion specific evolution of Mtb.

In summary, the fate of lesions diverged gradually from 4 wk postinfection onward, within the same host. Lesional heterogeneity arose, in part, through differential killing of bacteria after the onset of adaptive immunity, leading to a broad range of bacterial burden within and across animals. Many features of the rabbit model were reminiscent of observations made in nonhuman primates with active TB (Via et al., 2013; Lin et al., 2014; Martin et al., 2017): (1) individual lesions followed diverging trajectories; (2) a spectrum of pathology with occasional cavitation was observed across animals; (3) the average bacterial burden per lesion at 4 wk was homogeneous and around 10^4^ CFU, followed by a gradual broadening of the range of burden and immune killing as reflected by the “funnel" shape of CFU and CEQ kinetics at the lesion level; (4) the average bacterial burden per lesion decreased from 8 wk onward, and immune-mediated killing peaked between 12 and 16 wk; and (5) within the same host, different lesions harbored Mtb strains with different SNPs, supporting the concept of lesion-specific evolution of bacteria. Importantly, histology findings recapitulated observations made in human biopsies and resected tissues: Mtb bacilli were sparse in most caseous foci and, when present, were mostly associated with karyorrhectic neutrophils. This is consistent with neutrophils being increasingly recognized as providing a permissive site for Mtb replication (Diedrich et al., 2016; Mishra et al., 2017) (Lowe et al., 2012), and with the finding that Mtb is largely found in neutrophils in sputum, bronchoalveolar lavage, and cavity contents (Eum et al., 2010). The rabbit model thus reproduced several typical primate features and appeared as a suitable tool to evaluate drug activity in individual lung lesions.

### PZA markedly reduces bacterial burden in necrotic lesions

**Figure 3.**
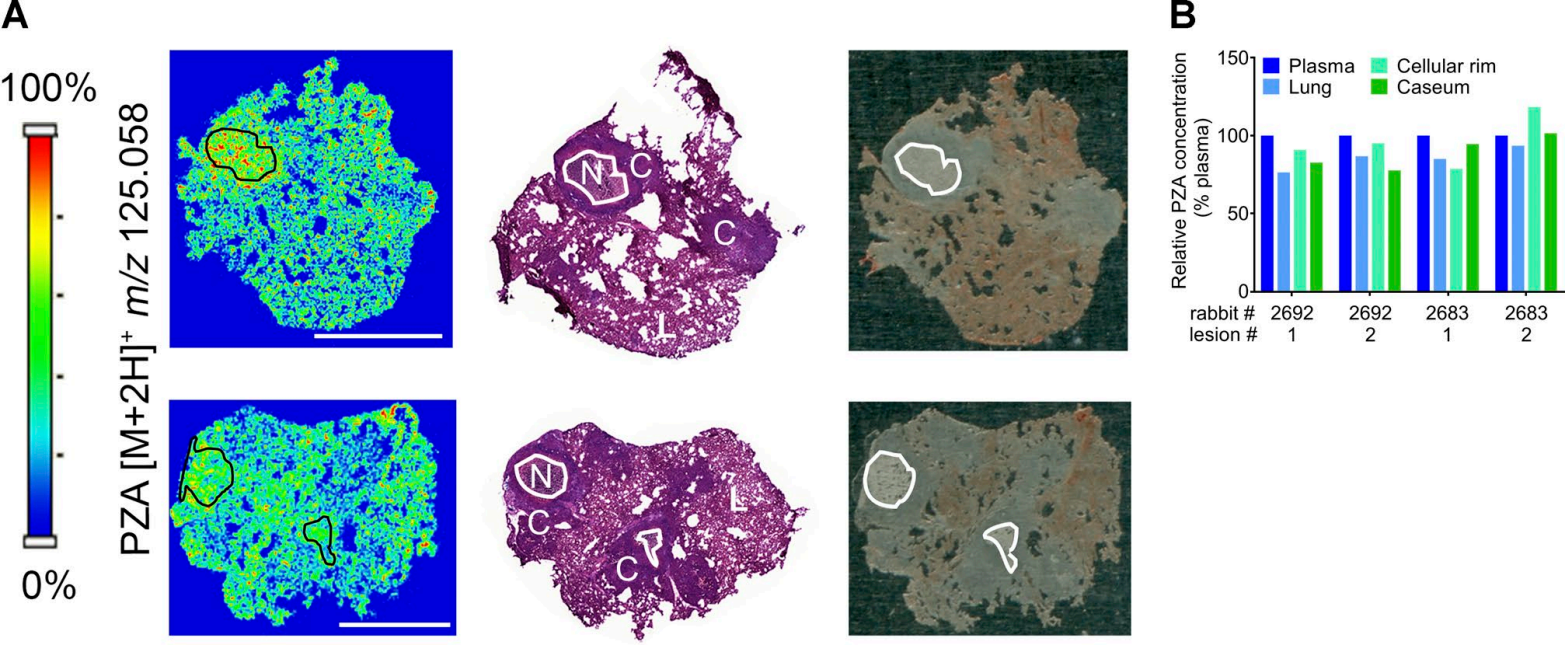
**Penetration of PZA in cellular and necrotic lesions. (A)** Two left panels: ion maps of PZA [M+2H]^+^ in rabbit lung tissue sections that encompass cellular and necrotic granulomas. The PZA [M+2H]^+^ signal intensity scale is linear and shown to the left. Middle panels: H&E stains of the corresponding adjacent sections. N, necrotic core or caseum; C, cellular rim or fully cellular granuloma; L, uninvolved lung. Right panels: optical images of the sections used for MALDI-MSI. The necrotic core of caseous granulomas is highlighted by the black contour lines in the ion maps (left) and white contour lines in the H&E stained section and optical image. Scale bars: 5 mm. **(B)** Quantitation of PZA in the necrotic or caseous core, cellular rim, and surrounding lung tissue of four lesions collected in two rabbits, obtained by LCM coupled to HPLC/tandem mass spectrometry. Each value was normalized to plasma concentrations measured at the time of tissue sampling.

PZA and rifampin are together responsible for almost all of the treatment shortening and sterilizing activity of the standard treatment of pulmonary TB (East African/British Medical Research Councils, 1972; Mitchison, 2004a), a finding that generally leads to the belief that PZA must sterilize necrotic lesions considered to harbor “persister” bacilli (Mitchison, 1985, 2010). However, efficacy studies in various animal models have delivered conflicted data. In published studies using guinea pig and mouse models, the effect of PZA on bacterial burden was assessed in whole lungs, and associations between sterilization and the presence of necrotic lesions were explored. This approach has delivered mixed conclusions as to the activity of PZA in necrotic/caseous lesions (Ahmad et al., 2011; Lanoix et al., 2015a; Irwin et al., 2016). Having shown key similarities of the rabbit model with human and nonhuman primate disease, we leveraged the model to directly quantify the lesion-centric activity of PZA and shed light on the pharmacodynamic basis of its sterilizing activity. CFUs and CEQs were measured in dissected lesions of PZA-treated and control rabbits after 5 and 10 wk of daily therapy at 175 mg/kg, starting 10 wk postinfection. To ensure on-target PZA exposure at steady-state, three rabbits were randomly assigned to a pharmacokinetic (PK) therapeutic drug monitoring substudy after 3 wk of treatment. The average area under the concentration-time curve (AUC) of PZA and pyrazinoic acid (POA; active metabolite of PZA) closely matched the simulated AUC based on PK in uninfected rabbits (Tables S3 and S4). Using matrix-assisted laser desorption ionization (MALDI) mass spectrometry imaging (MSI) and laser-capture microdissection (LCM) coupled to mass spectrometry, we also confirmed previous clinical observations by our group (Prideaux et al., 2015) and showed that in rabbits PZA distributes favorably from plasma into lung lesions (Fig. 3 A) and diffuses rapidly into necrotic foci to reach concentrations similar to those measured in plasma within 2 h postdose (Fig. 3 B). Thus, distribution to the site of infection and diffusion into avascular caseum does not constitute a barrier to PZA efficacy.

**Figure 4.**
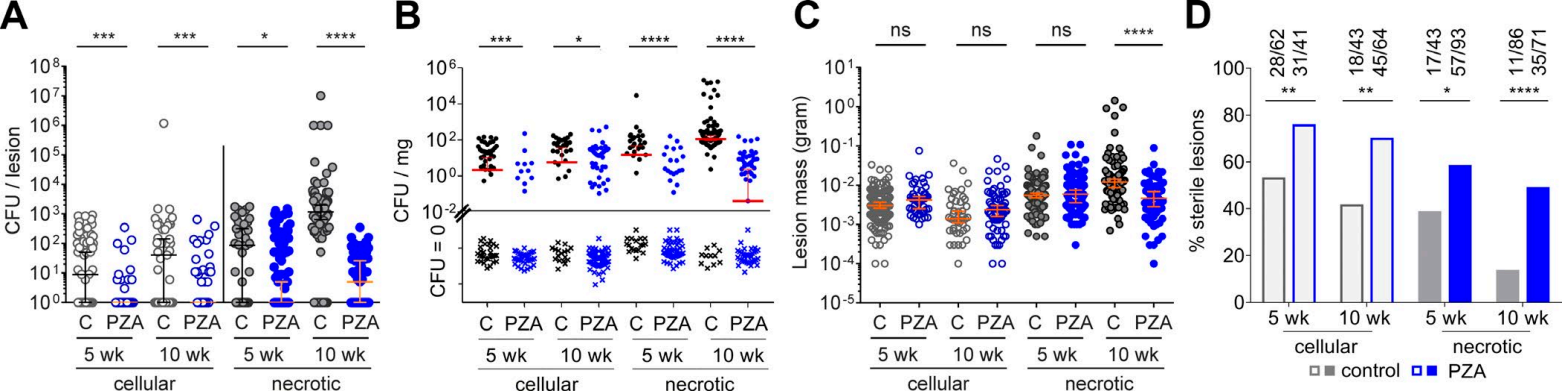
**Antimycobacterial activity of PZA in cellular and necrotic lesions. (A)** Effect of PZA on the bacterial burden of individual lesions in rabbits with active TB, after 5 and 10 wk of daily treatment. C, untreated controls. Median and 95% CI are shown (both median and 95% CI are at 10° for the PZA control groups in the cellular lesion category). Asterisks indicate statistical significance BY using the two-tailed Mann-Whitney *U* test. **(B)** Bacterial burden normalized to tissue weight in uninvolved lung tissue (“normal”) and in cellular and necrotic lesions. Because all CFU data are plotted on a log scale, all CFU values are converted to CFU + 1 order to include “zero CFU” data points. This method is not appropriate when normalizing to tissue weight because a difference of 1 would substantially skew the CFU-per-milligram dataset for the zero CFU subset. To visualize zero CFU data points, these were assigned an arbitrary low value of 10^−4^, then normalized to tissue weight. The median and 95% CI were calculated by using actual zero values. Medians that do not appear on the graph have a value of zero. **(C)** Effect of PZA treatment on lesion weight. Asterisks indicate statistical significance by using the Mann-Whitney *U* test. **(D)** Effect of PZA on the proportion of sterile lesions after 5 and 10 wk of treatment (actual numbers of sterile versus total lesions are indicated on top of each bar). Data from two independent experiments were combined to generate the plots. Statistically significant differences between two proportions were detected by using Fisher’s exact test. *, P < 0.05; **, P < 0.01; ***, P < 0.001; ****, P < 0.0001 (43 < *n* < 98 per treatment group).

At 175 mg/kg daily, PZA significantly reduced bacterial burden in cellular and necrotic lesions after 5 and 10 wk of treatment (Fig. 4 A). Likewise, CFUs normalized to lesion weight significantly decreased in all treated groups (Fig. 4 B). A significant PZA-mediated reduction of average lesion mass was observed only in the necrotic lesion category at the late time point (Fig. 4 C). This result indicates that PZA-mediated CFU reduction precedes a decrease of the average weight of necrotic lesions. PZA treatment also caused a significant increase in the proportion of sterile lesions, cellular and necrotic, after 5 and 10 wk of treatment, in addition to immune-mediated sterilization (Fig. 4 D), which peaks between 12 and 15 wk postinfection (Fig. 1 C). Thus, all observations were consistent with PZA being active in all lesion types and having at least as great an effect in necrotic granulomas as in cellular granulomas.

**Figure 5.**
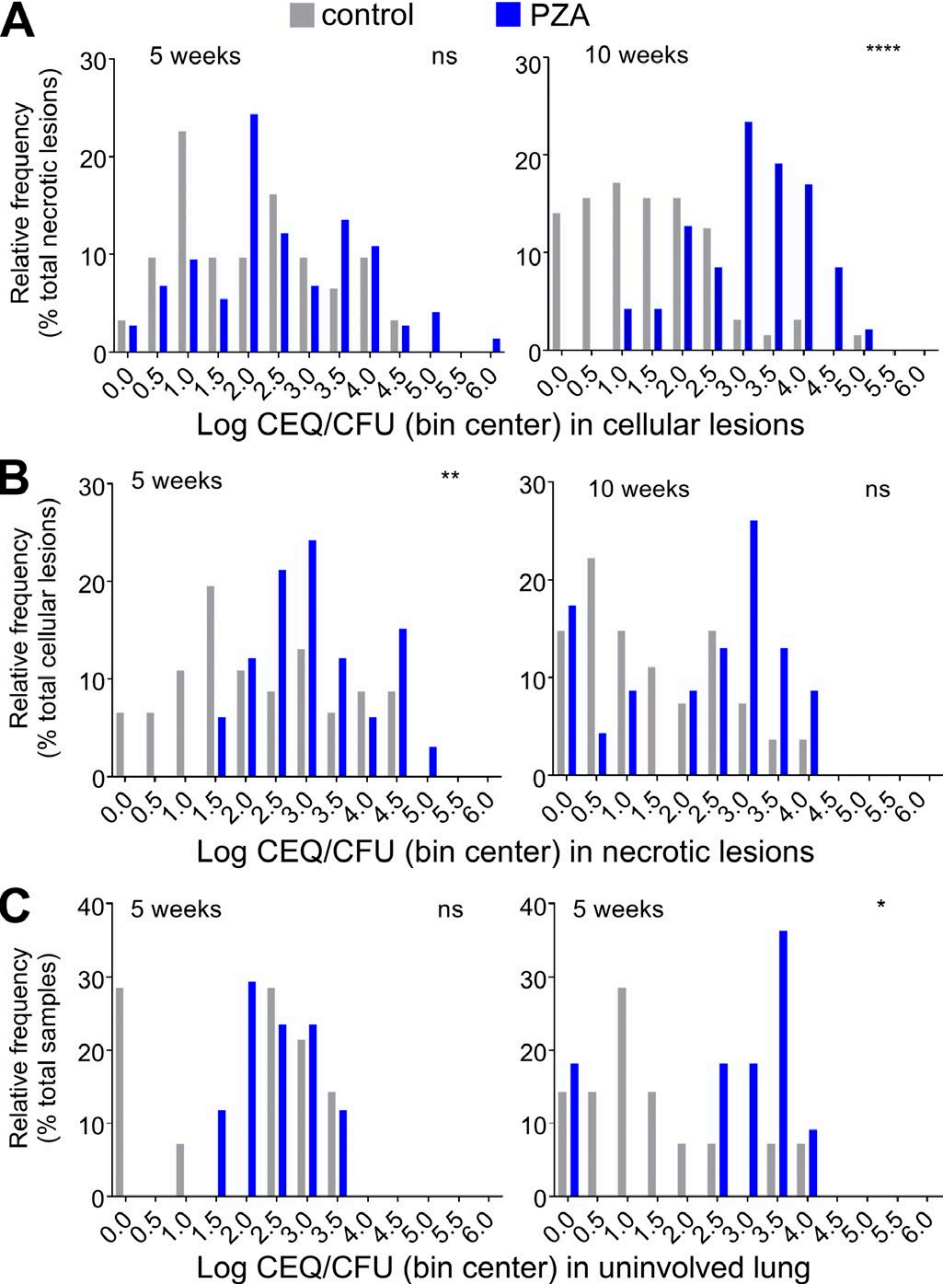
**Effect of PZA on the extent of bacterial killing or CEQ/CFU ratio at the lesion level in cellular lesions (A), necrotic lesions (B), and uninvolved lung (C).** To visualize the effect of PZA-mediated bacterial killing in addition to killing by the immune system, the data are presented as log CEQ/CFU ratio assigned to bins in 0.5-log increments in untreated rabbits (gray bars, immune killing only) and drug treated rabbits (blue bars, combined immune and PZA-mediated kill). Statistical analyses were performed by using nonlog normalized values. Data points where both CFU and CEQ values were below the LOD were excluded from this analysis. Data from two independent experiments were combined to generate the plots. Asterisks indicate statistically significant differences between the PZA treated and untreated groups by using the two-tailed Mann-Whitney *U* test (used to compare the CEQ/CFU ratios before log transformation and before binning). *, P < 0.05; **, P < 0.01; ***, P < 0.001; ****, P < 0.0001.

To distinguish the contribution of immune- and drug-mediated killing within individual lesions, we compared the CEQ/CFU ratios in treated and untreated rabbits as a function of lesion type (Fig. 5). Values of log CEQ/CFU per lesion were assigned to bins, and the proportion of lesions falling into each bin was plotted for treated and untreated lesions, categorized as cellular or necrotic. The same analysis was performed for uninvolved lung. This analysis revealed that in cellular lesions the contribution of PZA was not significant at 10 wk (Fig. 5 A) and that PZA most significantly added to immune-mediated killing in necrotic lesions after 10 wk of treatment (Fig. 5 B). By the time PZA treatment was initiated, most uninvolved lung samples had become sterile; however, PZA showed a clear effect at the late time point in uninvolved lung areas that were still culture positive (Fig. 5 C).

Thus, the PZA-mediated effect on CFUs, bacterial kill as reflected by CEQ/CFU, complete lesion sterilization, and lesion weight appeared most pronounced in necrotic lesions after 10 wk of treatment. This finding provides a pharmacodynamic explanation for the treatment-shortening property of PZA: the drug appears to exert its effect by targeting bacilli present in the difficult-to-sterilize caseum of necrotic lesions. Consistent with PZA’s activity in necrotic lesions, previous work by our group has shown that it is bactericidal against Mtb in an ex vivo caseum assay (Sarathy et al., 2018). Of note, the PZA concentration required to achieve a 1-log kill in this assay is around 20–40 µg/ml, levels that are reached only for a short portion of the dosing interval both in rabbits and in patients (Wilkins et al., 2006; Via et al., 2015b). The pH of ex vivo caseum under the assay conditions was around 7.5 and thus at the higher end of the pH spectrum we measured for rabbit caseum in the current study. Because PZA is more potent at a lower pH (McDermott and Tompsett, 1954; Salfinger and Heifets, 1988; Zhang et al., 2002), the ex vivo bactericidal assay may not have revealed the full potential of PZA in caseous environments. In the present study, the PZA dose of 175 mg/kg administered 7 d/wk reproduces human exposure at the lower end of the AUC spectrum observed in patients receiving the standard PZA dose of 1,500 mg daily (Wilkins et al., 2006). Therefore, we may have slightly underestimated the activity of PZA.

The marked efficacy of PZA in necrotic lesions in the rabbit model corroborates a comparative efficacy study in guinea pigs, which develop necrotic lesions and in which PZA was more active than in BALB/c mice, which do not present necrotic granulomas (Ahmad et al., 2011). In contrast, C3HeB/FeJ mice with large necrotic granulomas responded poorly to PZA treatment compared with C3HeB/FeJ mice that harbored mostly cellular lesions (Irwin et al., 2016). These discrepancies were attributed to the relatively high pH of C3HeB/FeJ mouse caseum (Lanoix et al., 2015a). The range of caseum pH measured in this study (6.1–8.0) is in line with the human caseum pH range of 5.5–8.0 (Koller and Leuthardt, 1934; Dahl, 1950; Weiss et al., 1954; Kempker et al., 2017). Thus, in rabbits and TB patients, different lesions present pH conditions that are more or less favorable to PZA’s activity, which may contribute to the observed interlesion variability in treatment response. It has also been suggested that localized areas of acute inflammation are associated with low O_2_ tension, accumulation of CO_2_ and lactic acid, and secretion of succinate by the tuberculous bacilli, leading to pH values as low as 5.0–5.5 in selected microenvironments (von Ardenne and Krüger, 1979; Watanabe et al., 2011).

There are a few limitations in this study. First, an apparent drop in average CEQs per lesion over time in cellular granulomas indirectly points to a potential decay of CEQs, possibly due to partial degradation of Mtb DNA by activated immune cells (Fig. S3, A and B). However, this apparent decrease could also be due to the emergence of new cellular lesions as disease progresses, with truly low cumulative burden. In addition, because DNA decay is likely to affect both untreated and PZA-treated lesion sets to the same extent, evaluation of PZA efficacy should remain largely unaffected. Second, the necrotic lesion category includes closed caseous nodules and open cavities, which cannot be distinguished at the time of dissection because large closed nodules and nascent cavities that have just connected to an airway cannot be differentiated upon macroscopic examination. Based on follow-up histological studies, we estimate that <5–10% of necrotic lesions are open cavities.

If PZA sterilizes necrotic lesions, why aren’t TB patients cured faster? First, PZA suffers from rather modest potency, with a minimum inhibitory concentration ranging from 12.5 to >100 µg/ml (∼1 mM), depending on pH and other environmental conditions (Zhang et al., 1999, 2002; Sarathy et al., 2018). Accordingly, PZA’s sterilizing effect in necrotic lesions was slow: in rabbits, the drug exerted most of its activity during the second half of the treatment period, after 5–10 wk (Fig. 5 B). Clinically, PZA is excluded from the continuation treatment phase (months 3–6) in patients with drug-susceptible TB based on (1) numerous large clinical trials conducted by the British Medical Research Council from which it was concluded that TB patients may not benefit from prolonged PZA therapy beyond 2 mo (Fox et al., 1999; Mitchison, 2004a) and (2) PZA-mediated hepatotoxicity (Centers for Disease Control and Prevention; American Thoracic Society, 2003). However, a trend toward lower relapse rates has been seen in trials in which PZA was administered beyond 2 mo, although statistical significance was not reached (Tuberculosis Service/British Medical Research Council, 1981; Mitchison, 2004a). In early bactericidal activity trials, PZA consistently shows modest activity during the first 2 d, after which the decrease in sputum counts accelerates, unlike other first-line drugs (Jindani et al., 2003). Likewise, a thorough analysis of serial sputum counts in patients receiving various combinations of front-line agents for 28 d revealed that the contribution of PZA was most pronounced during the second half of the study, from day 14 to 28 (Brindle et al., 2001). In addition, preclinical models have revealed a benefit of continuing PZA treatment beyond 2 mo, in the background of both first- and second-line regimens (Tasneen et al., 2008; Ahmad et al., 2013; Lanoix et al., 2015b). All these observations converge toward a rather slow “onset” of PZA activity. Together, our results may lead to rethinking the current treatment paradigm according to which PZA is given for the first 2 mo only. To avoid prolonged use of PZA beyond 2 mo given its hepatotoxicity, one could consider including PZA only after other agents have reduced the bacterial burden, leaving mostly persisters behind. Although clinical trials conducted by the British Medical Research Council in the 1960s to 1970s concluded no benefit of PZA beyond 2 mo, the utility of a late introduction of PZA has not been clinically tested, particularly in the context of today’s more refined readouts and biomarkers. Elucidation of the pharmacological mechanism underlying PZA’s unique clinical efficacy, together with the recent characterization of its antibacterial mechanism of action (Dillon et al., 2014; Gopal et al., 2016, 2017), provides the basis for the rational development of next-generation, more-potent PZA analogues.

## Materials and methods

### Infection, tissue dissection, and sample processing for bacterial burden

All animal studies were performed in Biosafety Level 3 facilities and approved by the Institutional Animal Care and Use Committee of the New Jersey Medical School, Rutgers University, Newark, NJ. Female New Zealand White rabbits (Millbrook Farm), weighing 2.2–2.6 kg, were maintained under specific pathogen-free conditions and fed water and chow ad libitum. The rabbits were infected with Mtb HN878 in rounds of six animals by using a nose-only aerosol exposure system as described, leading to the development of chronic active TB with cellular and necrotic granulomas in all animals, as well as occasional cavities (Subbian et al., 2011). 3 h postinfection, one rabbit from each round of infection was euthanized to determine the bacterial load implanted in the lungs, with a target of 1,000–3,000 CFU per rabbit. To characterize Mtb growth kinetics and pathology, untreated rabbits were necropsied and analyzed at the following time points: three, four, four, eight, seven, eight, and six rabbits at 4, 8, 10, 12, 15, 16, and 20 wk postinoculation, respectively. At each time point, rabbits were sedated with ketamine and xylazine, were euthanized by Euthasol, and underwent necropsy. The right and left lungs were removed and weighed. Individual lesions and uninvolved (not containing macroscopically visible lesions) lung tissue were dissected, weighed, and recorded; lesions were categorized as fully cellular (recorded and labeled as “cellular”) or necrotic (made of a cellular rim surrounding the necrotic or caseous core). Necrotic lesions are easily distinguishable from cellular granulomas due to (1) the presence of a white-to-yellow core visible upon macroscopic examination because granulomas are translucent and (2) the fact that cellular granulomas are more firm to the touch than necrotic lesions. When the distinction was subject to doubt, a fine needle was used to pierce through the center of the lesion, followed by gentle pressing leading to oozing of necrotic material in the case of necrotic lesions. The necrotic lesion category includes closed necrotic nodules and cavities, because it is not possible—upon macroscopic examination at the time of dissection—to reliably distinguish between a large closed nodule and a nascent cavity that has just connected to an airway. Special care was taken to remove the uninvolved lung tissue surrounding each lesion. An average of 10 necrotic lesions, 8 cellular lesions, and 6 uninvolved lung samples were collected from each rabbit. Tissue samples <0.05 g and >0.05 g were homogenized in either 200 µl or 500 µl PBS respectively. Serial dilutions of the tissue homogenates were prepared in PBS supplemented with 0.025% Tween and plated on Middlebrook 7H11 agar. The plates were incubated at 37°C for at least 21 d before determining final CFU counts. The lower LOD of CFU for each lesion is 3–5 depending on the total homogenization volume and the final volume and dilution plated (Dataset S1).

### Treatment

Starting 10 wk postinfection, 14 rabbits received 175 mg/kg PZA 7 d/wk for either 5 wk (7 rabbits) or 10 wk (7 rabbits). At 15 and 20 wk postinfection or after 5 and 10 wk of PZA treatment, rabbits were sedated with ketamine and xylazine, were euthanized by Euthasol, and underwent necropsy. Lesion and tissue samples were collected and processed as described above. After ∼3 and 7 wk of daily PZA treatment, blood was collected from the central ear artery of each rabbit predose and at 0.5, 1, 2, 4, 6, and 24 h after oral gavage to assess exposure in infected rabbits at steady-state and compare with the average exposure achieved in TB patients receiving a standard dose. Blood samples were centrifuged at 4,000 rpm for 5 min, and the supernatants (plasma) were transferred and stored at −80°C until analyzed by HPLC coupled to tandem mass spectrometry as described in (Via et al., 2015b).

### Human-equivalent PZA dose projection

PZA (Sigma Aldrich) was formulated in 40% sucrose and dosed at 175 mg/kg. The following rationale was followed in order to select this dose. In rabbits, the conversion of PZA to POA by the liver is significantly higher than in humans (Via et al., 2015b). Because POA is almost as potent as PZA against Mtb in vitro (Gopal et al., 2016), one could consider two different approaches in selecting a human-equivalent dose: (1) to match the sum of the AUCs of PZA and POA (AUC_PZA_ and AUC_POA_) achieved in TB patients receiving a standard dose of 1,500 mg or ∼450–500 mg/h/L, corresponding to a rabbit dose of 100 mg/kg, or (2) to match AUC_PZA_ only or 350–400 mg/h/L, corresponding to a rabbit dose of 275–300 mg/kg. The former approach assumes that circulating POA (as opposed to POA generated inside Mtb bacilli) is equipotent to PZA, which we found not to be the case in a separate study (Lanoix et al., 2016). Thus, we opted for a compromise and selected a dose of 175 mg/kg (Tables S3 and S4), leading to peak plasma concentrations (C_max_) of 50–75 mg/L (mean, 69.2 mg/L) and an AUC of 230–240 mg/h/L at steady-state, which correspond to the PZA C_max_ and AUC of TB patients at the higher (C_max_) and lower (AUC) end of the exposure spectrum, respectively (Wilkins et al., 2006).

### MALDI-MSI and LCM

Two rabbits were infected as described above, and disease was allowed to progress for 11 wk, at which point the rabbits received a single dose of PZA at 300 mg/kg (a higher dose was used than in efficacy studies to overcome the relatively poor ionization of PZA by MALDI-MSI), after which lesions and the surrounding lung tissue were collected 2 h postdose and stored as described previously (Zimmerman et al., 2017). 12-µm-thick tissue sections were prepared from γ-irradiated biopsies for MALDI-MSI and analyzed by using a MALDI LTQ Orbitrap XL mass spectrometer (Thermo Fisher Scientific) as previously described (Prideaux et al., 2015). Serial 25- and 12-µm-thick sections were prepared for LCM coupled to high-pressure liquid chromatography and tandem mass spectrometry quantitative analysis, and H&E staining respectively. Microdissection and quantification of caseum, cellular lesion, and uninvolved lung were performed as previously described (Zimmerman et al., 2017).

### Histology

Five pieces of lung tissue (one per lobe) from each rabbit were fixed in 10% formalin (Thermo Fisher Scientific) and sent to IDEXX BioResearch. These sections were paraffin-embedded and used for standard 5-µm sectioning. Sections were then stained with H&E for cellular composition and with Ziehl-Neelsen acid-fast staining to visualize Mtb bacilli. Images of the stains were captured and analyzed by using Pannoramic Viewer and Pannoramic Desk (Caliper Life Science).

### CEQ determination in lung and lesion tissues

Genomic DNA was extracted from homogenized tissue samples after Qiagen DNA extraction protocol by using the QIAamp 96 DNA Qiagen Kit (Slana et al., 2010). Samples were thawed, and 100 µl was transferred to a 96-well plate (S-Block; Qiagen) containing 180 µl stool lysis ASL buffer (Qiagen), 20 µl proteinase K (Qiagen), 1 µl reagent DX (Qiagen), and 250 µl of 0.1-mm zirconia-silica beads (MP Biomedicals) in each well. Tissues were digested for at least 1 h at 56°C, then inactivated for 1 h at 80°C. Samples were homogenized by two pulses of 1 min each at 1,750 rpm on the Geno/Grinder 2010 (Spex Sample prepP), with a 5-min break between pulses. The plate was centrifuged at 200 *g* to remove foam and precipitate beads, then loaded in a QIAcube HT (Qiagen). Samples were mixed with 600 µl of 50% buffer AL (Qiagen) and 50% ethanol (Fisher Scientific). 600 µl of supernatant from each well was transferred onto a 96-well plate silica column (Qiagen) and drawn through by using a vacuum pump. DNA samples were cleaned with three consecutive wash steps: 600 µl buffer AW1 (Qiagen), 600 µl AW2 (Qiagen), and 600 µl ethanol. DNA was then eluted with a total of 100 µl buffer AE (Qiagen). Mtb CEQ was quantified by using the previously described protocol (Muñoz-Elías et al., 2005) with sigF primer-probe combination (Integrated DNA Technologies) adapted from Lin et al. (2014). CEQ quantification was achieved by building standard curves by using serial dilutions of whole Mtb genome prepared from broth culture. qRT-PCR reactions were performed in duplicate at dilution 1×, 3×, and 9× (for a total of six quantitative PCR [qPCR] measurements per sample) on either the Mx3005P (Stratagene) or Step One Plus (Applied Biosystems) by using TaqMan Universal Master Mix II (Life Technologies).

### Validation of DNA extraction and qRT-PCR to assess CEQs in lung lesions

Bacteria were grown in 7H9 media with 10% ADC and 0.05% Tween 80. Serial dilutions of this culture were plated on 7H11 for CFU-per-milliliter enumeration (Fig. S3 C). To assess the total number of culturable, nonculturable, and dead cells in the same culture, all of which should be captured in qRT-PCR as CEQs, individual cells were counted with a hemocytometer (Neubauer) on a microscope (Leica DM6000 B, 400× magnification). The number of bacteria present in aggregates was estimated visually (Fig. S3 D). Serial dilutions were prepared, and 100 µl of each dilution was spiked in 100 µl of rabbit lung homogenate, then used as template for qRT-PCR as described above. To assess the variability of the qPCR procedure, each extract was diluted 1×, 3×, and 9× in duplicate to estimate the variability of the qPCR procedure and readout. The results are shown in Fig. S3 E. Next, we calculated the CEQ/total bacterial particles ratio in order to estimate the combined efficiency of DNA extraction and accuracy of qPCR analysis (Fig. S3 F). Under most conditions, the CEQ readouts ranged between 50 and 200% of the total estimated number of bacteria, thus a plus or minus twofold error. Precision was slightly lower at very low and very high bacterial loads, as expected. The LOD was ∼50 total bacterial cells, from which we inferred a limit of quantitation between 50 and 100 chromosomes.

### Whole-genome sequencing

After plating of rabbit lung tissue and lesion homogenates, mycobacterial genomic DNA was extracted as described previously (Blumenthal et al., 2010). Briefly, colonies were purified on 7H11 agar medium and used to inoculate 7H9 liquid cultures. Mtb HN878 genomic DNA was extracted as described (van Soolingen et al., 1991). DNA samples were prepared for sequencing by using the standard paired-end whole-genome DNA sample preparation kit from Illumina (Illumina Inc.). Samples were sequenced on an Illumina HiSeq 2500 next-generation sequencer, operated in paired-end mode, with a read length of 120 bp. The mean depth of coverage (reads per site) was 202.7× per genome (range, 90–1,445). Base-calling was performed by using Casava software, v1.8. The reads were aligned to Mtb HN878 (Genbank accession no. NZ_CM001043.1) as a reference genome by using the Burrows-Wheeler Alignment Tool (Li and Durbin, 2009). Indels were identified by building local contigs spanning sites with low coverage or heterogeneity of nucleotides, as described previously (Ioerger et al., 2010). Polymorphisms were identified by comparing (aligning) each genome to the sequence of HN878 and tabulating SNPs and insertions/deletions, excluding those in heterogeneous sites (majority nucleotide <70%) and sites in low-coverage (<10×) or repetitive regions. The laboratory stock of the parental strain HN878 was also resequenced, and differences shared among all the rabbit samples and the laboratory parental strain were also filtered out.

### Determination of caseum pH

About 30 min after euthanasia, the pH of pulmonary TB lesion caseum was determined by using different pH indicator strips (Table S1). Caseum was aseptically removed from incised lesions and applied as a thin layer on the indicator section of a pH strip that was prewetted with molecular-grade water (Life Technologies). The pH was read after 30 s of incubation. Surgical instruments were rinsed with molecular-grade water and dried between measurements. Up to three measurements of individual lesions were taken.

### Statistical analyses

This study included a total of 54 rabbits and 1,380 lesions. To detect statistically significant differences in CEQs, CFUs, or CEQs/CFUs per lesion, groups were compared by using a two-tailed Mann-Whitney *U* (nonparametric) test, which is adequate for analyzing nonnormally distributed (particularly “zero-inflated” in the case of CFU) datasets (GraphPad Prism 7). Proportions shown in Fig. 4 D were analyzed by using Fisher's exact test (two-sided for comparisons of treated versus untreated group pairs). In CEQ/CFU ratio analyses shown in Figs. 1, 5, and S2, lesions that harbored undetectable CEQs (the majority of which had no detectable CFUs) were excluded rather than set to zero because the limit of CEQ detection was ∼100 (Fig. S3). P values <0.05 were considered statistically significant. *, P < 0.05; **, P < 0.01; ***, P < 0.001; ****, P < 0.0001. All data are presented as median ± 95% confidence interval (CI), except for data shown as mean and standard deviation in Fig. S3 (C and D).

### Online supplemental material

Fig. S1 shows the characterization of lesion weight as a function of disease progression and bacterial burden; the bacterial burden normalized to tissue weight as a function of tissue and lesion type, the evolution of cumulative burden (CEQ) as disease progresses, and the relationship between bacterial burden and cumulative burden as disease progresses in cellular and necrotic lesions. Data shown in Fig. S1 are from drug-naive rabbits only. Fig. S2 shows histopathological evidence supporting release of cavity material into airways and the association of bacteria with neutrophils. Fig. S3. summarizes methodological considerations, validation, and calibration of the CEQ analyses. Table S1 summarizes pH measurements made in necrotic lesions and caseum from rabbits with active TB. Table S2 outlines the genetic polymorphisms of Mtb bacilli retrieved from rabbit lesions 20 wk postinfection. Table S3 describes the dose projection scenarios considered to reproduce human exposure of PZA in rabbits. Table S4 shows the PZA and POA PK parameters achieved in rabbits on treatment. Dataset S1 contains the complete CFU and CEQ data generated and analyzed in this work.

## Supplementary Material

Supplemental Materials (PDF)
