# Supplementary material for: Impact of immunopathology on the antituberculous activity of pyrazinamide
Source: J Exp Med. 2018 Aug 6;215(8):1975–86. doi: 10.1084/jem.20180518 (PMC6080910; doi:10.1084/jem.20180518)
Supplement: Supplemental Materials (PDF) [file JEM_20180518_sm.pdf]

Supplemental material

Blanc et al., https://doi.org/10.1084/jem.20180518

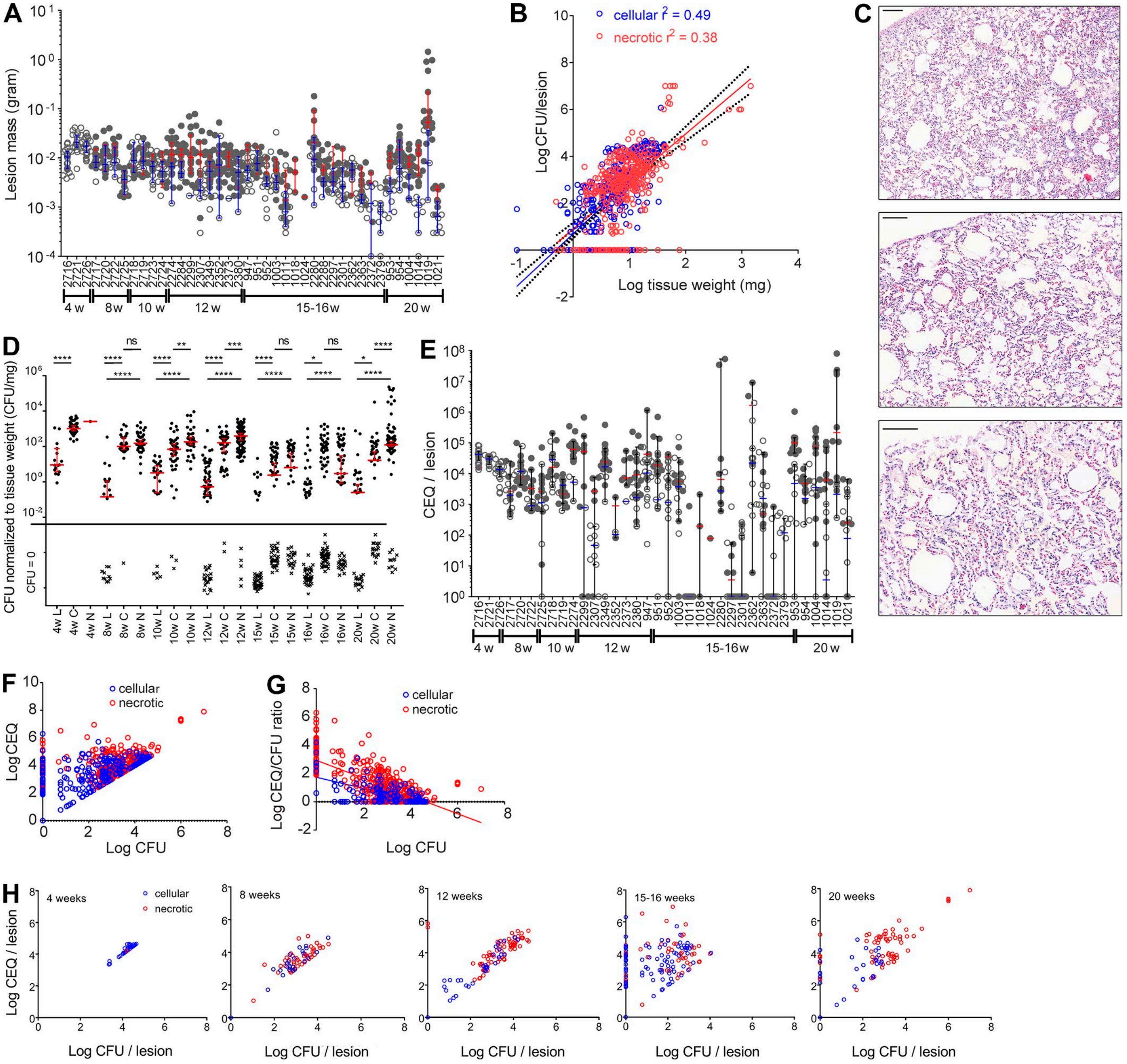

Figure S1. **Characterization of lesion weight, CFUs, and CEQs over the 20-wk disease course in drug-naive rabbits. (A)** Range of lesion weight per rabbit over the 20-wk disease course. Median and 95% CI are shown separately for cellular (open circles and blue median bars) and necrotic (closed circles and red median bars) lesions in each rabbit. **(B)** Plot of CFUs per lesion as a function of lesion weight. Cellular and necrotic lesions collected from 4 to 20 wk postinfection were included in the analysis. R^2 = 0.49 for cellular lesions and 0.38 for necrotic lesions. The mean regression lines (continuous line) and error (dotted lines) are shown. **(C)** H&E staining of typical uninvolved lung tissue pieces collected for CFU and CEQ enumeration. Scale bars: 100 µm. **(D)** Bacterial burden normalized to tissue weight in uninvolved lung tissue (L) and in cellular (C) and necrotic (L) lesions from 4 to 20 wk postinfection. Because all CFU data are plotted on a log scale, all CFU values are converted to CFU + 1 order to include "zero CFU" data points. However, this method is not appropriate when normalizing to tissue weight because a difference of 1 would substantially skew the CFU per milligram dataset for the zero CFU subset. To visualize zero CFU data points, these were assigned an arbitrary low value of 10^{-4} and then normalized to tissue weight. The median and 95% CI were calculated by using actual zero values. Medians that do not appear on the graph have a value of zero. **(E)** Distribution of cumulative burden per lesion (CEQ/lesion) within individual animals, analyzed from 4 to 20 wk postinfection. Median and 95% CIs are shown separately for cellular (blue bars) and necrotic (red bars) lesions in each rabbit. **(F)** Lesion-centric CEQs as a function of bacterial burden in untreated rabbits, plotted for cellular (blue) and necrotic (red) lesions, showing the overall higher bacterial burden in necrotic lesions and the higher spread of cumulative burden (CEQs) in lesions with low than with high bacterial load. As CFUs increase, the spread of CEQs decreases, as shown by the overall tapered shape of the cloud. **(G)** Plot showing the inverse correlation trend between the extent of killing (CEQ/CFU ratio) and the bacterial burden within individual lesions, from 4 to 20 wk postinfection, indicating failed immunity in lesions with high burden. **(H)** CEQs in cellular and necrotic lesions as a function of bacterial burden over time, showing a burst of immune-mediated kill between 12 and 16 wk postinfection. A "tail" of necrotic lesions with high bacterial burden and CEQ = CFU is seen at 20 wk postinfection, suggesting failed immunity.

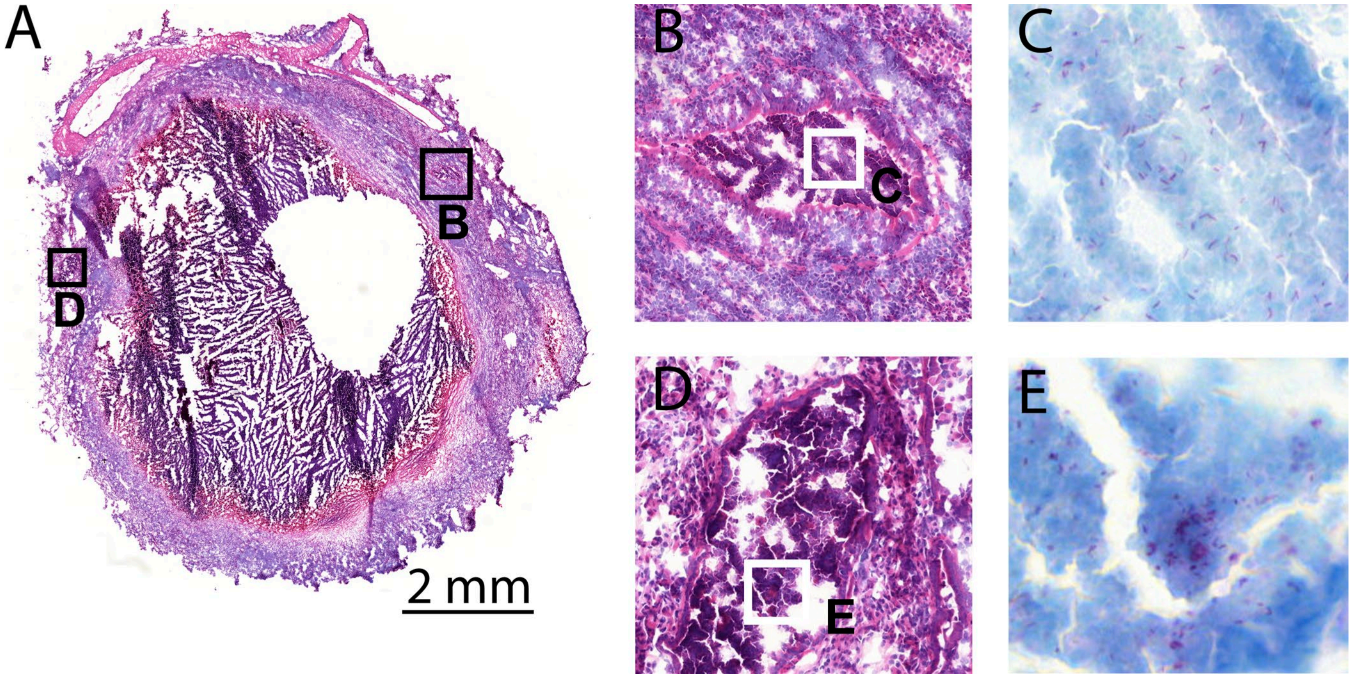

Figure S2. **Histopathological evidence supporting release of cavity material into airways. (A)** H&E staining of a frozen lung section from a large rabbit cavity highlighting two small bronchiole airways in the periphery of the cellular and fibrotic cuff. **(B and D)** Magnification of the two regions highlighted in A showing the two bronchioles filled with neutrophils and caseous debris. **(C and E)** Ziehl-Neelsen stains and magnification of the regions highlighted by the white contour lines in B and D. High numbers of acid-fast bacilli are present in association with neutrophils in the bronchiole lumen.

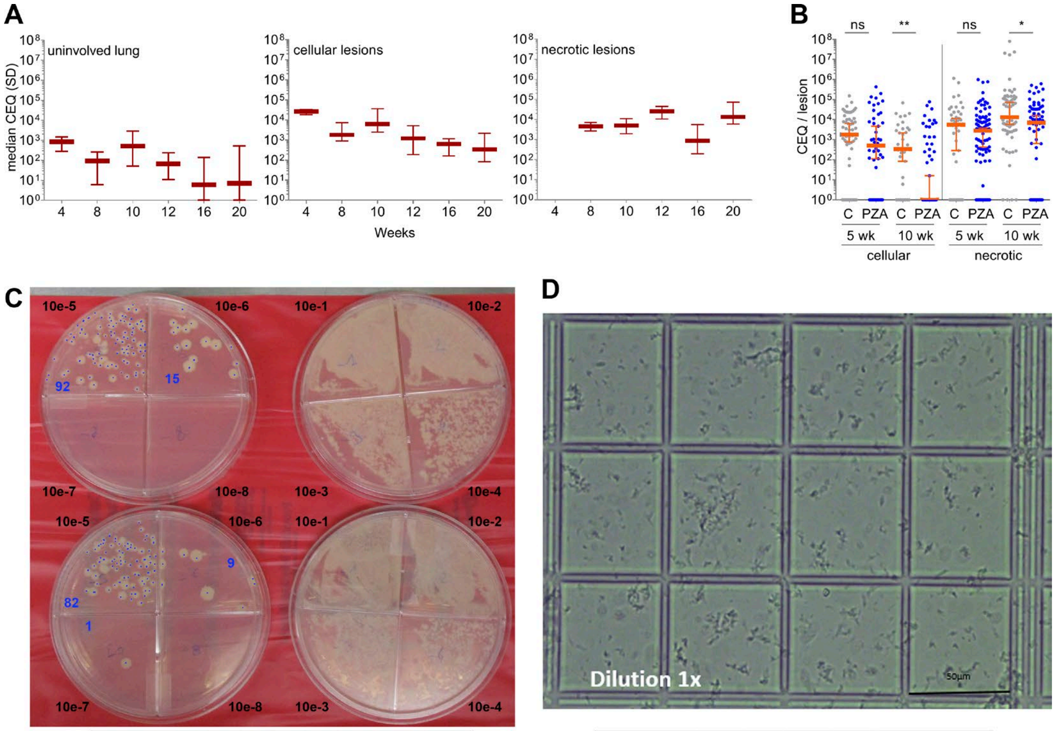

Dilution (log)	-3	-4	-5	-6	-7	-8	Average
#1 100 μL	x	x	96	9	1	0	**8.9x10^7 CFU/ml**
#2 100 μL	x	x	82	15	0	0	

Count on 10 squares of 2500μm^2	#1	#2	#3	#4	Average
10 x Dilution	140	95	86	104	**4.1x10^8 bacteria/ml**

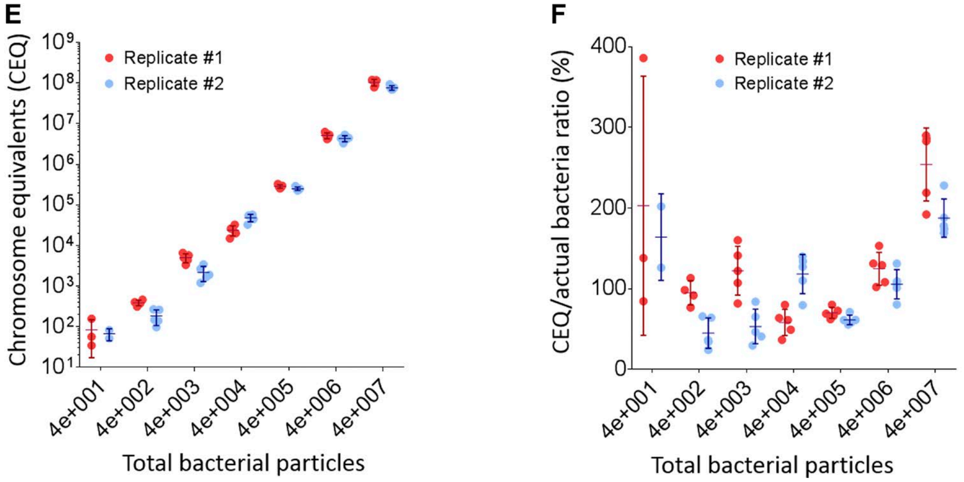

Figure S3. **CEQ analyses: methodology, pitfalls and calibration. (A)** Evolution of CEQs per lesion, a surrogate of cumulative burden, over the entire course of the experiment in uninvolved lung, cellular lesions, and necrotic lesions. The trend over time suggests partial decay of CEQs in uninvolved lung and cellular lesions. The average CEQs in uninvolved lung and cellular lesions, but not in necrotic lesions, decreased slightly from 10 to 20 wk reflecting a disconnect between CEQs and cumulative burden, which could be explained by a partial loss of CEQ; median and 95% CI are shown. **(B)** CEQs per lesion measured in PZA-treated and control rabbits after 5 and 10 wk of treatment in cellular and necrotic lesions. In PZA-treated rabbits, the average CEQs per lesion decreased between 5 and 10 wk of treatment in cellular but not in necrotic lesions. Thus, it appears that a loss of CEQs is observed in cellular lesions where bacteria mostly reside in macrophages but not—or at least to a lesser extent—in necrotic lesions where the bulk of the bacterial load is extracellular in caseum. In addition, as shown in Fig. S1, in uninvolved lung samples of untreated animals, we found a substantial burden up to 12 wk postinfection, after which the majority of uninvolved lung areas became sterile whereas the average CEQ/CFU ratio was low around 1. **(C–F)** Validation and reproducibility of DNA extraction and CEQ measurements. **(C)** Plating of bacterial culture used for the CEQ calibration and validation. CFU counts are recorded in the table appended below the image. **(D)** Individual bacterial cell counts by using a hemocytometer (Neubauer) resulting in the total count of culturable (CFUs), viable but nonculturable, and dead bacteria. Bacterial particles were counted in 10 squares of 2,500 µm^2 in quadruplicate. **(E)** qPCR (see Materials and methods for details) results showing the dynamic range up to 10^8 bacteria per sample. The x axis shows the total number of bacteria as counted in D. **(F)** Combined efficiency of DNA recovery and qPCR expressed as the ratio between CEQs and total bacteria counted in D. CEQ readouts are within plus or minus twofold of actual bacterial numbers (50–200% or target).

Table S1. **pH of necrotic lesions and caseum from rabbits with active TB**

Rabbit ID	Lesion type	Lung lobe	pH	pH (paper type)	Water added?	Comments
2583	Caseous granuloma	RUL	6.5	4.0–7.0 (EMD Chemicals Inc.)	No	1/2 lesion placed directly onto paper
2583	Caseous granuloma	RUL	6.5		No	1/2 lesion placed directly onto paper
2583	Caseous granuloma	LLL	6.5		No	1/2 lesion placed directly onto paper
2583	Caseous granuloma	RLL	6.1		No	1/2 lesion placed directly onto paper
2743	caseum	ND	7	4.5–10.0 (Whatman type CF)	No	Caseum spread over the three sections of pH paper
2743	caseum	ND	8	0.0–13.0 (Hydrion)	Yes	Caseum spread over the three sections of pH paper with 1 droplet of water
2743	caseum	ND	6.5		Yes	Caseum vortexed for 30 s in small beaded tubes, then added to pH paper at 50 µL volume
2751	caseum	ND	6.5	4.5–10.0 (Whatman type CF)	Wetted pH paper strip	Caseum evenly spread over the three sections of pH paper
2749	caseum	LUL	6.8	6.5–10.0 (MColorpHast; Millipore) and 4.0–7.0 (Special Indicator; Millipore)		Caseum applied like soft butter is spread with a knife
2749	caseum	LML	7.1, 6.8			
2749	caseum	LLL	6.8, 7.1			
2749	caseum	LLL	7.1, 6.8			
2749	caseum	RUL	6.8, 6.8, 7.0			
2749	caseum	RLL	6.8, 6.8			
2749	caseum	RLL	6.8, 6.5			
2749	caseum	RLL	6.8, 6.5			
2749	caseum	RLL	6.8, 6.5			
2749	caseum	LUL	6.8			

Whole necrotic lesions or caseum were collected from rabbits with active TB. Whole lesions were sectioned in two halves, and one half was placed on pH paper as indicated. Caseum was scooped out of cavities or large necrotic granulomas and spread across the three sections of prewetted pH paper. Multiple pH values reported in one row represent multiple measurements (sometimes with different pH papers) with caseum extracted from the same lesion. Dissection instruments were washed with molecular grade water and wiped with a dry paper towel between measurements to avoid contamination with basic disinfectant. RUL, right upper lobe; RLL, right lower lobe; LUL, left upper lobe; LML, left middle lobe; LLL, left lower lobe; CF, color fixed.

Table S2. **Summary of genetic polymorphisms found in 23 lesions from four rabbits infected with Mtb HN878 for 20 wk**

Rabbit ID	Lesion	Treatment	Organ	Granuloma type	Polymorphisms
**953**	**T#10**	**Control**	**Lung**	**Necrotic**	
953	T#12	Control	Lung	Necrotic	
953	T#13	Control	Lung	Necrotic	Rv2024c:T1367S
954	T#05	Control	Lung	Necrotic	
954	T#20	Control	Lung	Necrotic	Rv0159c/PE3:-A (-1bp del in Ala238), Rv1878/glnA3:A238S, Rv3794/embA:A571T
953	T#20	Control	Lung	Cellular	Rv0355c/PPE8:P1326P, Rv3386:G114G
953	T#21	Control	Lung	Cellular	
954	T#07	Control	Lung	Cellular	Rv2827c:A42T
954	T#12	Control	Lung	Cellular	
954	T#13	Control	Lung	Cellular	
948	T#10a	PZA	Lung	Necrotic	
948	T#10b	PZA	Lung	Necrotic	
948	T#30	PZA	Lung	Necrotic	
949	T#18	PZA	Lung	Necrotic	
948	T#01	PZA	Lymph node	NA	
948	T#02a	PZA	Lymph node	NA	
948	T#02b	PZA	Lymph node	NA	
948	T#03	PZA	Lymph node	NA	Rv2482c/plsB2:L725L,Rv3331/sugI:Y128C
948	T#04	PZA	Lymph node	NA	
948	T#16a	PZA	Lung	Cellular	
948	T#16b	PZA	Lung	Cellular	
948	T#21a	PZA	Lung	Cellular	
948	T#21b	PZA	Lung	Cellular	
HN878 Reference strain					Input inoculum used to infect rabbits

Lesions were obtained from four different rabbits as color coded.

Table S3. **Dose projection scenarios in rabbits compared to human exposure**

Dose (mg/kg)	AUC_{PZA} (ng/h/ml)	AUC_{POA} (ng/h/ml)	AUC_{PZA} + AUC_{POA} (ng/h/ml)	Comments
**Rabbits**				
100	133,890	332,040	465,930	1. Matching sum of PZA and POA AUC
125	167,560	416,710	584,260	
150	200,830	498,060	698,900	
175	234,300	581,070	815,380	Compromise of 1 and 2
200	267,780	664,090	931,860	
225	301,250	747,100	1,048,300	
250	334,720	830,110	1,164,800	
275	368,190	913,120	1,281,300	
300	401,670	996,130	1,397,800	2. Matching AUC of PZA alone
325	435,140	1,079,100	1,514,300	
350	468,610	1,162,100	1,630,800	
375	502,080	1,245,200	1,747,200	
400	535,550	1,328,200	1,863,700	
425	569,030	1,411,200	1,980,200	
450	602,500	1,494,200	2,096,700	
475	635,970	1,577,200	2,213,200	
500	669,440	1,660,200	2,329,700	
**TB patients**				
1,500	400,000	76,000	476,000	

Simulations are based on the rabbit PK model described in Via et al., 2015. A dose of 175 mg/kg was chosen to achieve a compromise between matching the sum of PZA + POA AUC in humans (= 476 µg/h/ml) corresponding to a rabbit dose of 100 mg/kg, or matching PZA AUC only (= 400 µg/h/ml) corresponding to a rabbit dose of 300 mg/kg. At 175 mg/kg (green shade), the observed C_{max} in rabbits (mean 70 µg/ml [Table S4]) lies in the upper quartile of the human C_{max} range, and the PZA AUC lies at the lower end of the clinical exposure range (Wilkins et al., 2006).

Table S4. **PZA and POA PK parameters achieved in rabbits receiving daily oral doses of 175 mg/kg (at steady state) compared to the target AUC based on PK simulations described in Table S3**

	PZA 175 mg/kg (po)	POA
	**Mean (SD); *n* = 3**	
$AUC_{[0\text{-}24]}$ (µg/h/ml)	236.7 (33.3)	583.0 (149.2)
C_{max} (µg/ml)	69.2 (8.4)	70.4 (17.3)
T_{max} (h)	1.33 (0.58)	3.33 (1.15)
Target AUC based on PK simulations (µg/h/ml)	234.3	581.1

References

Via, L.E., R. Savic, D.M. Weiner, M.D. Zimmerman, B. Prideaux, S.M. Irwin, E. Lyon, P. O'Brien, P. Gopal, S. Eum, et al.. 2015. Host-mediated bioactivation of pyrazinamide: Implications for efficacy, resistance, and therapeutic alternatives. ACS Infect. Dis. 1:203–214. doi:10.1021/id500028m

Wilkins, J.J., G. Langdon, H. McIlleron, G.C. Pillai, P.J. Smith, and U.S. Simonsson. 2006. Eur. J. Clin. Pharmacol. 62:727–735. doi:10.1007/s00228-006-0141-z
